# Hospitals

**Published:** 1877-11

**Authors:** 


*Hospitals: Their History, Organization and Construction. Boylston Prize Essay of Harvard University for* 1876. By W. Gill Wylie, M. D. New York: D. Appleton and Company, 549 & 551 Broadway. 1877. Buffalo: Herger and Ulbrich. pp. 236.

This is not an exhaustive history but notices in a few words the most famous and expensive hospitals of Europe and the United States. The origin and partial development of the idea dates backward to the Buddhists of India, several hundred years before the Christaian era. "It is said that the sick were treated in the temples of Æsculapius, 1134 years B. C., at Titanus, a city of Peloponnesus, but there is no evidence that these temples were used for the purpose of treating the sick poor; still they were in some respects similar to our hospitals." India, Persia and Rome may claim honor for originating the thought, but to the teachings of Christ is due the quickening influence which is making the full development of that thought a grand and world wide power for the alleviation of human suffering.

The body of this volume is devoted to the consideration, elucidation and application of principles upon which depend the purest, safest, most efficient and comfortable hospital accommodations and service; together with ground plans of a large number of hospital buildings, an ideal model, and the proposed plan of the Johns Hopkins Hospital of Baltimore.

The authors points of criticism of the structures noticed, are well sustained. The book will commend itself to all persons especially interested in these matters and is also suggestive with regard to the sanitary construction of all houses. The author strongly favors Miss Nightingale's one story pavilion plan, with ridge ventilation, open fires, and hot water pipes as accessories in heating. The minutiæ of hospital construction and furnishing are carefully considered. Hospital infection and diseases are regarded as inevitable under certain well known and commonly existing conditions, and the author shows how by wise construction and administration, they may be placed at a minimum or done away with altogether. M. B. M.

